# Ventricular growth, measured by cardiac MRI, is not different in patients with tetralogy of fallot versus pulmonary atresia with intact ventricular septum or critical pulmonary stenosis after right ventricular outflow tract reconstruction

**DOI:** 10.1186/1532-429X-16-S1-O106

**Published:** 2014-01-16

**Authors:** Jon Detterich, Abraham Kaslow, Jay D Pruetz, John Wood

**Affiliations:** 1 Cardiology, Children's Hospital Los Angeles, Los Angeles, California, USA; 2 Keck School of Medicine, at the University of Southern California, Los Angeles, California, USA

## Background

Cardiac MRI is used to measure right ventricular end diastolic volume indexed to body surface area (RVEDVi) and ejection fraction (EF) in the setting of pulmonary insufficiency (PI). There is data describing optimum RVEDVi, in tetralogy of fallot (TOF) patients, to provide a competent pulmonary valve and maintain long term RV function. Pulmonary atresia with intact ventricular septum (PAIVS) and critical pulmonary stenosis (CPS) are congenital heart defects with variable right ventricle (RV) morphology including severe hypoplasia. A subset of these patients undergo initial palliation with right ventricular outflow tract reconstruction to allow for antegrade flow across the pulmonary valve and PI to encourage RV growth over time. There is limited RV volumetric and function data to guide pulmonary valve replacement in this population. The lack of data concerning RV growth in this population may lead to mistimed valve replacement such that RV systolic or diastolic function is permanently compromised. Hypothesis: RVEDVi growth will be different between patients with TOF and PAIVS/CPS; and systolic function will be decreased at equivalent volumes in the PAIVS/critical PS group.

## Methods

Two groups of patients were studied for RV size by MRI volumetric measurement: Group 1 were patients with TOF s/p repair and residual PI, Group 2 was comprised of patients with PAIVS or CPS with HRV who underwent RVOT reconstruction. MRI was performed on GE or Philips 1.5T scanners at Children's Hospital Los Angeles. Short axis cine images were analyzed for RV and LV size. Phase contrast imaging was used to assess flow in the main and branch pulmonary arteries to determine regurgitant fraction. Student's T-test or Wilcoxon Rank Sum tests were used to compare age, height, weight, BSA, RV and LV sizes between the two groups. Linear regression was performed with the model RVEDVi vs. age at time. Multivariate regression was performed with the outcome variable RVEF.

## Results

**Figure 1:**
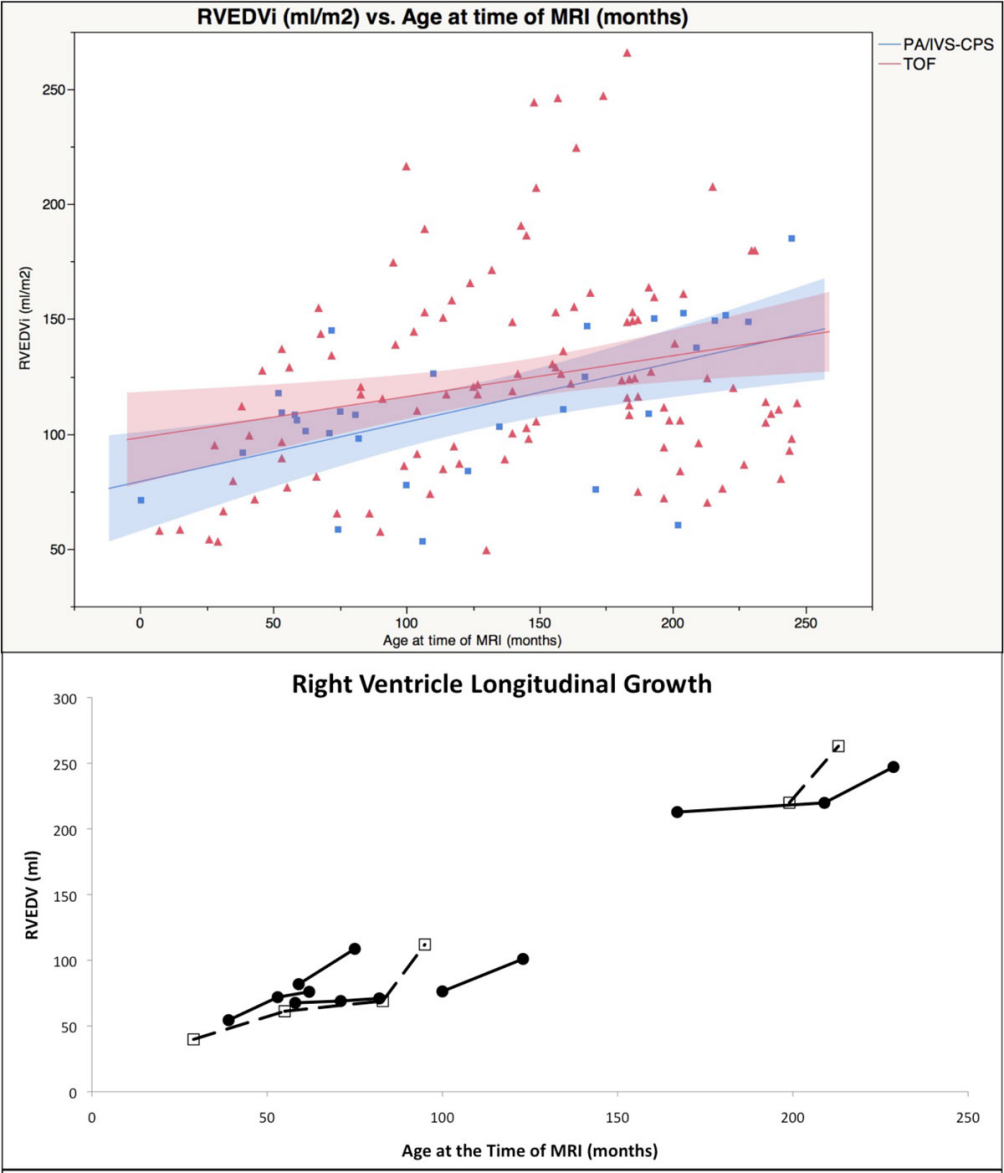
**The top panel demonstrates RVEDVi vs Age utilizing cross-sectional data for two patient groups: Tetralogy of Fallot s/p repair and PA/IVS or critical pulmonary stenosis with hypoplastic right ventricle s/p RV outflow tract reconstruction**. The lower panel shows longitudinal growth in a subset of patients for which data was available.

114 patients with TOF and 25 patients with PA/IVS-CPS had data reviewed spanning a period of 14 years. The groups were well matched for height, weight and BSA, while there was a trend toward younger age in the PAIVS group (P = 0.06). The RVEDVi, RVESVi and RVEF of patients with PA/IVS-CPS and TOF were not significantly different (P = 0.12, 0.15, and 0.49 respectively). RVEDVi was plotted against age and there was no difference in slope of the regression lines, suggesting similar growth of the RV between PAIVS-CPS and TOF, which was consistent with limited longitudinal data (Figure 1). RV regurgitant fraction, LVEF and RV:LV EDV ratio were independent predictors of RVEF in both groups (Table 1).

**Table 1:** Multivariate Analysis of RVEF

TOF	Parameter Estimate	Model R ^2 ^	Prob > |t|
RV regurgitant fraction	0.459	0.58	< 0.0001
LV ejection fraction	0.601		< 0.0001
RV:LV end diastolic volume	-21.43		< 0.0001
PA/IVS- CPS			
RV regurgitant fraction	0.203	0.62	< 0.0001
LV ejection fraction	0.684		0.0003
RV:LV end diastolic volume	-16.67		0.0017

Results of multivariate linear regression for RVEF in TOF patients and PA/IVS-CPS patients.

## Conclusions

RV growth and systolic function is similar between a PA/IVS-critical PS group and TOF group after RV outflow tract reconstruction. There is significant RV:LV interaction driving RV systolic function in both groups.

## Funding

There was no funding support for this study.

